# Differences in sexual behaviour and sexual practices of adolescents in Nigeria based on sex and self-reported HIV status

**DOI:** 10.1186/1742-4755-11-83

**Published:** 2014-12-06

**Authors:** Morenike O Folayan, Morolake Odetoyinbo, Brandon Brown, Abigail Harrison

**Affiliations:** Department of Child Dental Health, Obafemi Awolowo University, Ile-Ife, Nigeria; Institute of Public Health, Obafemi Awolowo University, Ile-Ife, Nigeria; Positive Action for Treatment Access, Lagos, Nigeria; Program in Public Health, Department of Population Health & Disease Prevention, University of California, Irvine, USA; Department of Behavioral and Social Sciences, School of Public Health, Brown University, Providence, RI 02912 USA

**Keywords:** Adolescents, Nigeria, HIV, Sex, Practices, Behaviour

## Abstract

**Background:**

Sexual behaviour and sexual practices affect the risk for acquisition and transmission of HIV infection. This study tries to identify differences in sexual behaviour (condom use with non-marital partners, multiple sexual partnerships transactional sex and age mixing in sexual relationships), sexual practices (oral, anal and vagina sex), and forced sexual initiation based on sex and HIV status of adolescents in Nigeria.

**Method:**

Face to face interviewer-administered questionnaires were used to collect information from a nationally representative sample of 10–19 years old adolescents residing in Nigeria. Data included information on age of sexual debut, sexual behaviour and sexual practices. Association between HIV status, sex, sexual behaviour and sexual practices, and predictors of use of condoms during the last vaginal sexual intercourse were determined.

**Result:**

More self-reported HIV positive than HIV negative females had experienced forced sexual initiation (p = 0.008). Significantly more female than male adolescents had engaged in transactional sex (p < 0.001) and had sex with partners who were older than them by 10 years or more (p < 0.001). Vaginal (95.2%), oral (26.6%) and anal (7.8%) sex were practiced by male and females irrespective of HIV status. More females reported oral sex (p = 0.001). Being a female (p = 0.001), having genital itching in the last 12 months (p = 0.04)and having engaged in anal sex in the last 12 months (p = 0.009) reduced the odds of using a condom at last vaginal intercourse. Having a HIV positive or negative status did not significantly increase the odds of using a condom at last vaginal intercourse.

**Conclusion:**

Differences in sexual behaviour and sexual practices of adolescents was observed based on sex and not on HIV status. History of forced sex initiation however differed by HIV status. Tailored interventions for male and female adolescents are required to reduce their risk of HIV infection. Tailored interventions are also required for adolescents living with HIV to improve their sexual and reproductive health.

## Background

Estimates show that nearly 50% of the 35.3 million people living with HIV acquired infection before the age of 25 years through sexual transmission[1]. The 2012 HIV statistics in Nigeria showed that approximately 20% of males and 37% of females 10 – 19 years old had commenced sexual intercourse[2]. In effect, adolescents in Nigeria do show evidence of early sex initiation[3].

Continued engagement with sexual intercourse may be modified by a variety of factors including one’s HIV status. Studies from Uganda suggest that sexual behaviour and practices among adolescents living with HIV (ALHIV) did not differ significantly from what was observed in the general population[4]. A large number of adolescents initiate sex early with associated low use of condom and contraceptives. The most common reason given for current use of condoms was prevention of pregnancy (57.0%). Although Birungi[4] observed that condom use amongst ALHIV was similar to that observed among the general population, there is no evidence suggesting this behaviour will be observed in other countries.

Data from Nigeria show that adolescents 15 to 19 years old engage in high sexual risk behaviour: 56.4% of sexually active boys and 39.6% of sexually active girls had had unprotected sex with non-marital sexual partners in the last 12 months of a survey[3]. The proportion of adolescents who engage in this high risk sexual behaviour was higher than what was observed in other age groups. Other high risk behaviours – transactional sex, multiple sex partnership, age mixing of sexual partners - are on the increase among adolescents in Nigeria[5].

The association between HIV infection and rape has also been highlighted[6, 7]. Reports have shown that 4-6% of all adolescent girls in south-western Nigeria experience rape[8, 9]. A prior study had highlighted the need for public dialogue on the association between HIV and rape in Nigeria and how to prevent rape[10].

There is currently little known about factors that drive choice of sexual practices and sexual behaviours in ALHIV. Evidence is required to inform the design of interventions that will help improve the quality of lives ALHIV, including reducing their risk for STI and HIV super-infection.

This study is one effort in that direction. We try to identify differences in use of condoms with non-marital partners, multiple sexual partnerships, transactional sex and age mixing in sexual relationships, forced sexual initiation, and sexual practices (vagina, oral and anal sex),based on differences in sex and HIV status of adolescents in Nigeria.

## Methods

This study reports on findings from a large nationally diverse cross-sectional survey of adolescents aged 10–19 years conducted in Nigeria between July and October 2012. The sample size was derived from a best estimate of the number of adolescents living with HIV based on the 2010 HIV sero-sentinel survey[11]. Crude estimates project that approximately 268,000 adolescents aged 10 – 19 years were living with HIV in Nigeria in the year 2012. A minimum of 660 interviews were needed per study arm to provide a 95% confidence interval with an estimated dropout rate of 10%.

The study was conducted in 12 States in Nigeria and the Federal Capital Territory. The 12 states were randomly selected from the 18 states that hosted the first 25 HIV treatment sites in Nigeria. Two states were randomly selected from each geopolitical zone in Nigeria. The selected states were Lagos, Oyo, Imo, Enugu, Edo, Rivers, Kaduna, Kano, Borno, Adamawa, Plateau and Benue.

Recruitment of ALHIV was primarily through contact with parents who were HIV positive and were attending support group meetings in the study sites. Networks of persons living with HIV in the various study states were contacted, study was introduced and their interest in the study solicited. Contacts were also made with physicians of ALHIV at the various treatment centres in the study sites, to obtain their support to help introduce and refer adolescents who were interested in participating in the study. Contact details of study team in each state were given to physicians working with ALHIV, support group leaders and network of persons living with HIV contacts. Interested participants were to contact study team members.

Recruitment of in- and out-of-school was done in youth centres within the vicinities where HIV positive adolescents were recruited. It was assumed that based on the national HIV prevalence of 3.4%[12], a large number of in- and out-of-school adolescents would be HIV negative. Many may also not know their HIV status since the rate of HIV testing in the country was very low: only about 15% of Nigerians had had a HIV test by the end of 2011[13].

The study depended on self-reporting of HIV status by participants. It was not feasible to conduct HIV tests at the point of questionnaire administration for several reasons. First, 10–14 year old children would require parental consent and individual assent for HIV test to be conducted. In view of the low uptake of HIV counselling and testing (HCT), and the high level of HIV stigma in communities in Nigeria[14], there was high risk of low HCT uptake during the study. Second, the feasibility of conducting HIV tests at the multiple diverse recruitment venues in line with the national HCT testing guidelines would have been logistically challenging and beyond the scope of this study’s resources to address.All adolescents’ age 10-19years were eligible to participate in the study. Informed consent was sought from adolescent 16 years and above. Parental informed consent and assent was sought for adolescents 10 to 15 years. Any adolescent who was living with AIDS, managing chronic debilitating diseases like cancer, or was mentally challenged were excluded from participating in this study.

Data was collected from each adolescent in a private room using a face to face interviewer administered structured questionnaire. Data on age, sex, self-reported HIV status, use of alcohol and psychoactive substances were collected. Assessment of the sexual and reproductive health needs was done using a questionnaire validated for use for the 2007 national HIV and AIDS reproductive health survey (NARHS)[12]. For the purpose of this study, the questionnaire was adapted to capture information on vagina, anal and oral sex practices. Other questions in the questionnaire captured details on sexual behaviour such as multiple sexual partnering, unprotected sex, transactional sex and sex with persons who were 10 years older. Knowledge of HIV transmission was measured using five questions that asked if the following was a risk factor for HIV transmission: sexual intercourse, sharing of share of sharp unsterilized objects, transfusion with unscreened blood, sharing of unsterilized needles and transmission of infection from mother to unborn child. Knowledge of HIV prevention was assessed using eight questions namely: One can reduce risk of contracting HIV by having sex with only one faithful uninfected partner, by using condoms, by abstaining from sex, by delaying sexual debut, by avoiding sex with sex workers, by reducing the number of sexual partner, by avoiding sex with people with multiple sex partners, and by avoiding sharing of sharp objects.

Key words/phrases (including sensitive ones) for each selected community were translated during training of study assistants. The semi-translated survey tool served as a reference document when working in the field. A similar approach was successfully used for the 2005 and 2007 NARHS[12, 15], as well as the 2007 and 2010 Integrated Biological and Behavioural Surveillance Surveys[16, 17] conducted in Nigeria.

Some data were transformed for ease of analysis. Age was grouped into 10–14 years and 15–19 years. Respondents’ HIV status was classified into four groups: adolescents who self-report as negative adolescents, adolescents who self-report as HIV positive; adolescents who were known to be HIV positive based on recruitment method but self-reported as HIV status unknown; and adolescents who self-report status as untested.

Scores on knowledge about HIV transmission, and HIV prevention were summed in order to calculate final knowledge scores. In order to dichotomize the variables, the mean of the final scores on knowledge about HIV transmission, and knowledge about HIV prevention served as cut-off points respectively. Those who had scores equal to or above the cut-off points were categorised as having good knowledge. Those who had scores below the cut-off point were categorised as having poor knowledge.

Bivariate analysis was conducted to determine association of HIV status and risky behaviours (having multiple sexual partners, unprotected sex, transactional sex and sex with persons who were 10 years older). Dependent variables were sexual practices (anal, vagina and oral sex), sexual behaviour (age of sexual debut, history of condom use, having multiple sex partnership, age of sex partner), and history of forced sexual initiation. Independent variables were sex and HIV status. All associations that were significant were fitted into a binomial logistic regression model to determine predictors of condom use during anal, oral and vagina sex. Pearson or Fischer’s Exact Chi-Square was used to test significance of associations between variables. Comparison of continuous variables (age) was done using t-test. Statistical significance was defined at P < 0.05 with a 95% confidence interval. Analysis was conducted using STATA version 12.0.

Ethics approval for the study was obtained from the National Institute of Medical Research Institutional Review Board, the Health Research Ethics Committee of Plateau State and Health Research Ethics Committee of the Federal Capital Territory, Abuja.

## Results

### Study population

A total of 1,601 adolescents completed the study questionnaire. Only 1,574 questionnaires were considered complete enough for data entry. The sample population was 18% more than the minimum sample size (N = 1,320) required for the study.

Of the 749 HIV positive study participants recruited, 434 (57.9%) self-reported as HIV positive, nine (1.2%) self-reported as HIV negative, 43 (5.8%) stated they did not know their HIV status, and 263 (35.1%) gave no response on HIV status. Of the 825 adolescents recruited in the public places, 142 (17.2%) reported HIV negative HIV test result, 19 (2.3%) reported a positive HIV status, 619 (75.0%) stated they did not know their status and 45 (5.5%) gave no response. Overall, 151 (9.6%) participants self-reported as HIV negative, 453 (28.8%) as HIV positive, and 970 (61.6%) did not know their HIV status or did not respond to the question on HIV status.

#### Use of alcohol and psychoactive drugs

The majority of the respondents did not report drinking alcohol (77.0%) or using psychoactive drugs (94.7%). See Table 1. There were no significant difference observed in the number of HIV positive male and female (p = 0.19), and HIV negative male and female (p = 0.33) adolescents who used psychoactive substances.

**Table 1:** **Sexual and HIV profile of respondents by self reported HIV status and sex (N = 1574)**

Variables	HIV status												Total (%) N = 1574
	Self-reported HIV Negative N = 151			Self-reported HIV status unknown (N = 969)						Self-reported HIV positive N = 454			
				Untested (N = 663)			Recruited as HIV positive (N = 306)						
	Male n = 68	Female n = 83	P value	Male n =368	Female n =295	P value	Male n =134	Female n =172	P value	Male n =221	Female n = 233	p value	
**History of sexual intercourse**													
Yes	32 (47.1)	39 (47.0)		90 (24.5)	62 (21.0)		16 (11.9)	27 (15.7)		78 (35.3)	92 (39.5)		**436 (27.7)**
No	36 (52.9)	44 (53.0)	0.99	278 (75.4)	233 (79.0)	0.30	118 (89.1)	145 (84.3)	0.35	143 (64.7)	141 (60.5)	0.36	**1138 (72.3)**
**Mean age (± SD) at sexual debut**													
Mean age	15.48 ± 1.8	15.15 ± 1.9	0.50	14.84 ± 2.2	14.00 ± 1.9	0.03	14.87 ± 2.0	12.94 ± 3.2	0.053	15.42 ± 1.6	14.76 ± 1.6	0.01	**14.79 ± 2.0**
**Intake of alcohol**													
Yes	23 (33.8)	20 (24.1)		77 (20.9)	74 (25.1)		26 (19.4)	35 (20.3)		53 (24.0)	54 (23.2)		**362 (23.0)**
No	45 (66.2)	63 (75.9)	0.21	291 (79.1)	221 (74.9)	0.23	108 (81.6)	137 (79.7)	0.89	168 (76.0)	179 (76.8)	0.84	**1212 (77.0)**
**Use of psychoactive drugs**													
No drug use	66 (97.1)	76 (91.6)		348 (94.6)	278 (94.2)		130 (97.0)	169 (98.3)		209 (94.6)	215 (92.3)		**1491 (94.7)**
Use of drug(s)	2 (2.9)	7 (8.4)	0.14	20 (5.4)	17 (5.8)	0.86	4 (3.0)	3 (1.9)	0.70	12 (5.4)	18 (7.7)	0.33	**93 (5.5)**
**History of STI**													
Yes	42 (61.8)	45 (54.2)		180 (48.9)	152 (51.5)		61 (45.5)	90 (50.3)		125 (56.6)	118 (50.6)		**813 (51.7)**
No	26 (38.2)	38 (45.8)	0.93	188 (51.1)	143 (48.5)	0.50	73 (54.5)	82 (49.7)	0.24	96 (43.4)	115 (49.4)	0.21	**761 (48.3)**
**Knowledge of HIV transmission**													
Good	47 (69.1)	70 (84.3)		152 (41.3)	119 (40.3)		32 (23.9)	30 (17.4)		113 (51.1)	133 (57.1)		**696 (44.2)**
Poor	21 (30.9)	13 (15.7)	*0.03	216 (58.7)	176 (59.7)	0.80	102 (76.1)	142 (82.6)	0.16	108 (48.9)	100 (42.9)	0.20	**878 (55.8)**
**Knowledge of HIV prevention**													
Good	52 (76.5)	65 (78.3)		177 (48.1)	157 (53.2)		22 (16.4)	28 (16.3)		117 (52.9)	133 (57.1)		**751 (47.7)**
Poor	16 (23.5)	18 (21.7)	0.79	191 (51.9)	138 (46.8)	0.19	112 (83.6)	144 (83.7)	0.97	104 (47.1)	100 (42.9)	0.38	**823 (52.3)**
**Awareness about condom**													
**Yes**	44 (64.7)	59 (71.1)		212 (57.6)	179 (60.7)		80 (59.7)	108 (62.8)		147 (66.5)	143 (61.4)		**972 (61.8)**
**No**	24 (35.3)	24 (28.9)	0.47	156 (42.4)	116 (29.3)	0.43	54 (40.3)	64 (37.2)	0.58	74 (33.5)	90 (38.6)	0.25	**602 (38.2)**
**Knowledge of where to obtain condom**													
**Yes**	9 (13.2)	17 (20.5)		43 (11.7)	30 (10.2)		29 (21.6)	25 (14.5)		30 (13.6)	29 (12.4)		**212 (13.5)**
**No**	59 (86.8)	66 (79.5)	0.24	325 (88.3)	265 (89.8)	0.54	105 (78.4)	147 (85.5)	0.11	191 (86.4)	204 (87.6)	0.72	**1362 (86.5)**

### Age of sexual debut, knowledge of HIV and awareness about condom

These were 791 (50.3%) males and 783 (49.7%) females who contributed sexual health data. Only 436 (27.7%) of respondents gave a history of having had sex (Table 1).

#### Age of sexual debut

The average age of sexual debut for the study population was 14.8 ± 2.0 years. There was a significant difference in the age of sexual debut of female and male adolescents who self-reported as HIV positive: females had a lower age of sexual debut than males (14.8 yrs vs 15.4 yrs; p = 0.01). There was no significant difference in the age of sexual debut of female and male adolescents who self-reported as HIV negative (15.2 yrs vs 15.5 yrs; p = 0.50), female adolescents who self-reported as HIV positive or HIV negative (14.8 yrs vs 15.2 yrs; p = 0.20), and male adolescents who self-reported as HIV positive or HIV negative (15.4 yrs vs 15.5 yrs; p = 0.54).

#### Knowledge of HIV transmission and HIV prevention

From our measures, 696 (44.2%) respondents had good knowledge of HIV transmission while 751 (47.7%) had good knowledge of HIV prevention. More female than male adolescents who self-reported as HIV negative had good knowledge of HIV transmission (84.3% vs 69.1%; p = 0.03). There was no significant difference in the percentage of male and female adolescents who self-reported as HIV positive who had good knowledge of HIV transmission (51.1% vs 57.1%; p = 0.20). See Table 1. More male adolescents who self-reported as HIV negative had good knowledge of HIV transmission than males who self-reported as HIV positive (69.1% vs 51.1%; p = 0.009). Also, more female adolescents who self-reported as HIV negative had good knowledge of HIV transmission than females who self-reported as HIV positive (84.3% vs 57.1%; p < 0.001).

There was no significant difference in the proportion of male and female adolescents who self-reported as HIV negative (76.5% vs 78.3%; p = 0.79) and those who self-reported as HIV positive (52.9% vs 57.1%; p = 0.38) who had good knowledge of HIV prevention. See Table 1. More female adolescents who self-reported as HIV negative had good knowledge of HIV prevention than females who self-reported as HIV positive (78.3% vs 57.1%; p < 0.001). Also, more male adolescents who self-reported as HIV negative had good knowledge of HIV prevention than males who self-reported as HIV positive (76.5% vs 52.9%; p = 0.001).

#### Awareness about condoms

972 (61.8%) respondents had heard about or seen a condom. Of these, 212 (21.8%) knew where to obtain a condom. There was no significant difference observed in the percentage of male and female adolescents who self-reported as HIV positive (66.5% vs 61.4% P = 0.25) and those who self-reported as HIV negative (64.7% vs 71.1% P = 0.47) who were aware about condoms. There was also no significant difference in the percentage of male and female adolescents who self-reported as HIV positive (13.6% vs 12.4% P = 0.72) and those who self-reported as HIV negative (13.2% vs 20.5% P = 0.24) who knew where to obtain a condom.

### History of sex and sexual behaviour

Table 2 shows the profile of the sexual behaviour of the 216 (49.5%) male and 220 (50.5%) female respondents who had ever had sexual intercourse. Responses below are based on this sub-sample of sexually active youth.

**Table 2:** **History of sex and sexual behavior of respondents by self reported HIV status and sex**

Variables	HIV Status												Total (%)
	Self-reported HIV Negative n = 71			Self-reported unknown HIV Status (n = 159)						Self-reported HIV Positive n = 170			N = 436
				Untested (n = 152)			Recruited as HIV positive (nN = 43)						
	Male n = 32	Female n = 39	P value	Male n =90	Female =62	P value	Male n =16	Female n = 27	P value	Male n =78	Female n = 92	p value	
**Reason for sexual debut**													
Love	7 (21.9)	17 (43.6)		21 (23.3)	16 (25.8)		4 (25.0)	7(25.9)		19 (24.4)	26 (28.2)		**117 (26.8)**
Having fun	13 (40.6)	3 (7.7)		37 (41.1)	3 (4.8)		4 (25.0)	1 (3.7)		23 (29.5)	10 (10.9)		**94 (21.5)**
Peer pressure	9 (28.1)	9 (23.1)		27 (30.0)	13 (21.0)		4 (25.0)	5 (18.5)		31 (39.7)	19 (20.7)		**117 (26.8)**
For money	0 (0.0)	1 (2.5)		0 (0.0)	4 (6.5)		0 (0.0)	0 (0.0)		0 (0.0)	8 (8.7)		**13 (3.0)**
Forced	1 (3.1)	9 (23.1)	0.001	5 (5.6)	24 (38.7)	0.000	1 (6.3)	10 (37.0)	0.05	5 (6.4)	26 (28.2)	0.000	**81 (18.5)**
Others	0 (0.0)	0 (0.0)		0 (0.0)	0 (0.0)		0 (0.0)	0 (0.0)		0 (0.0)	3 (3.3)		**3 (0.2)**
No response	2 (6.3)	0 (0.0)		0 (0.0)	2 (3.2)		3 (18.7)	4 (14.9)		0 (0.0)	0 (0.0)		**14 (3.2)**
**History of transactional sex**													
Yes	2 (6.3)	11 (28.2)		7 (7.8)	21 (33.9)		5 (31.3)	6 (22.2)		11 (14.1)	26 (28.2)		**89 (20.4)**
No	30 (93.7)	28 (71.8)	0.03	83 (92.2)	41 (66.1)	0.000	11 (68.7)	21 (77.8)	0.51	67 (85.9)	66 (71.8)	0.03	**347 (79.6)**
**History of concurrent multiple sexual partnerships**													
Yes	9 (28.1)	17 (43.6)		41 (44.6)	42 (67.7)		9 (56.3)	10 (37.0)		33 (42.3)	33 (35.9)		**194 (44.5)**
No	23 (71.9)	22 (56.4)	0.18	49 (55.4)	20 (32.3)	0.007	7 (43.7)	17 (63.0)	0.22	45 (57.7)	59 (64.1)	0.39	**242 (55.5)**
**History of sex in last 12 months**													
Yes	26 (81.2)	30 (76.9)		71 (78.9)	52 (83.9)		14 (87.5)	16 (59.3)		69 (88.5)	79 (85.9)		**357 (81.9)**
No	6 (18.8)	9 (23.1)	0.66	19 (21.1)	10 (16.1)	0.44	2 (12.5)	11 (40.7)	0.09	9 (11.5)	13 (14.1)	0.62	**79 (18.1)**
^**+**^ **Form of sexual intercourse ever**													
Anal	2 (6.3)	2 (5.1)	1.00	3 (3.1)	4 (6.5)	0.44	1 (6.3)	2 (7.4)	1.00	9 (11.5)	6 (6.5)	1.00	**29 (6.7)**
Oral	10 (31.3)	6 (15.4)	0.11	19 (21.1)	20 (32.3)	0.12	3 (18.7)	5 (18.5)	1.00	29 (37.2)	23 (25.0)	1.00	**115 (26.4)**
Vagina	26 (81.3)	30 (76.9)	0.66	64 (71.1)	51 (82.3)	0.12	14 (87.5)	15 (55.5)	0.05	66 (84.6)	78 (84.8)	0.05	**344 (78.9)**
**Age of last sexual partner**													
Same age	14(43.8)	5 (12.8)		32 (35.6)	9 (14.5)		2 (12.5)	2 (7.4)		29 (37.2)	18 (19.6)		**111 (25.5)**
≥ 10 years older	1 (3.1)	8 (20.5)		1 (1.1)	7 (11.3)		1 (6.3)	7 (25.9)		7 (9.0)	18 (19.6)		**50 (11.5)**
< 10 years younger	3 (9.3)	0 (0.0)	0.001	13 (14.4)	0 (0.0)	0.000	1 (6.3)	0 (0.0)	0.000	0 (0.0)	1 (1.1)	0.03	**18 (4.1)**
Do not know	14 (43.8)	26 (66.7)		44 (48.9)	46 (74.2)		12 (75.0)	18 (66.7)		42 (53.8)	55 (59.7)		**257 (58.9)**

^+^multiple responses possible.

#### Reason for sexual debut

The main reasons for sex debut were love (26.8%) and peer pressure (26.8%). Forced sex (18.5%) was the third main reason for sexual debut. Significantly more female than male adolescents reported forced sexual initiation (31.4% vs 0.05%; p <0.001). Also, significantly more female adolescents who self-reported as HIV positive had experienced forced sex when compared to female adolescents who self-reported as HIV negative (28.2% vs 23.1%; p = 0.008).

#### History of transactional sex

Significantly more female than male adolescents had engaged in transactional sex (29.1% vs 11.6%; p < 0.001). There was no significant difference in the percentage of female adolescents who self-reported as HIV positive or HIV negative who had engaged in transactional sex (28.2% vs 28.2%; p = 0.70).

#### Multiple concurrent sexual partnerships

One hundred and ninety four (44.5%) sexually active respondents had had multiple concurrent sexual partners. There was no significant difference in the percentage of male and female adolescents who self-reported as HIV positive (42.3% vs 35.9%; p = 0.39), and the percentage of male and female adolescents who self-reported as HIV negative (28.1% vs 43.6%; p = 0.18) who had multiple concurrent sexual partners. Also, there was no significant difference in the percentage of male adolescents who self-reported as HIV positive or HIV negative (42.3% vs 28.1%; p = 0.16), and the percentage of female adolescents who self-reported as HIV positive or HIV negative (35.9% vs 43.6%; p = 0.41) who have multiple concurrent sexual partners.

#### Forms of sexual practice

The majority of the respondents (78.9%) practiced vaginal sex. Oral and anal sex was practiced by 26.4% and 6.7% of respondents respectively. There was no significant difference in the percentage of male and female adolescents who self-reported as HIV positive who had had anal sex (11.5% vs 6.5%; p = 1.00), and the percentage of male and female adolescents who self-reported as HIV negative who had had anal sex (6.3% vs 5.1%; p = 1.00). Also, here was no significant difference in the percentage of male adolescents who self-reported as HIV positive or HIV negative who had had anal sex (11.5% vs 6.3%; p = 0.40), and the percentage of female adolescents who self-reported as HIV positive or HIV negative who had had anal sex (6.5% vs 5.1%; p = 0.79).

#### Age of last sexual partner

The majority (58.9%) of the respondents were not aware of the age of their last sexual partner. This was commoner with female than male adolescents (65.9% vs 51.5%; p = 0.003). Among those who knew the age of their sex partners, more female than male adolescents engaged in sex with partners who were 10 years or more older than them during their last sexual act (18.2% vs 4.6%; p < 0.001). There was no significant difference in the percentage of female adolescents who self-reported as HIV positive or HIV negative who had had sex with partners 10 years or more older than them (19.6% vs 20.5%; p = 0.90).

### HIV sexual risk profile of adolescents

Table 3 shows the HIV risk profile of the 357 (22.7%) respondents who had had sex in the last 12 months of the survey.

**Table 3:** **HIV risk profile of respondents who had been sexually active in the last 12 months by self reported HIV status and sex**

Variables	HIV status												Total (%) N = 357
	HIV negative n = 56			unknown HIV Status (N = 153)						HIV Positive n = 148			
				Untested (n = 123)			Known HIV positive (n = 30)						
	Male n = 26	Female n =30	P value	Male n =71	Female n = 52	P value	Male n =14	Female n =16	P value	Male n =69	Female n = 79	p value	
^**+**^ **Forms of sexual intercourse in the last 12 months**													
Anal	2 (7.7)	2 (6.7)	1.00	3 (4.2)	3 (5.8)	0.70	1(7.1)	2 (12.5)	1.00	9 (13.0)	6 (7.6)	0.28	**28 (7.8)**
Oral	10 (38.5)	6 (20.0)	0.13	13 (18.3)	15 (28.8)	0.17	3 (21.4)	5 (31.3)	0.69	23 (33.3)	20 (25.3)	0.54	**95 (26.6)**
Vagina	26 (100.0)	30 (100.0)	-	64 (90.1)	48 (92.3)	0.68	13 (92.9)	15 (93.8)	1.00	66 (95.7)	78 (98.7)	0.34	**340 (95.2)**
^**+**^ **Combination of sexual routes for sexual intercourse**													
Anal only	0 (0.0)	0 (0.0)	-	0 (0.0)	0 (0.0)	-	0 (0.0)	0 (0.0)	-	2 (2.9)	1(1.3)	0.60	**3 (0.8)**
Oral only	0 (0.0)	0 (0.0)	-	0 (0.0)	0 (0.0)	-	0 (0.0)	0 (0.0)	-	0 (0.0)	0 (0.0)	-	**0 (0.0)**
Vagina only	16 (61.5)	24 (80.0)	0.10	11 (15.5)	0 (0.0)	*0.002	11 (78.6)	10 (62.5)	0.44	38 (55.1)	55 (69.6)	0.07	**165 (46.2)**
Anal + oral	0 (0.0)	0 (0.0)	-	0(0.0)	0 (0.0)	-	0 (0.0)	0 (0.0)	-	1 (1.4)	0 (0.0)	0.47	**1 (0.3)**
Anal + oral + vagina	2 (7.7)	2 (6.7)	1.00	1 (1.4)	2 (3.8)	0.57	1 (7.1)	2 (12.5)	1.00	6 (8.7)	5 (6.3)	0.76	**21 (5.9)**
Oral + vagina	8 (30.8)	4 (13.3)	0.19	2 (2.7)	3 (5.8)	0.65	2 (14.3)	3 (18.8)	1.00	22 (31.9)	18 (22.8)	0.46	**62 (17.4)**
Anal + vagina	0 (0.0)	0 (0.0)	-	0 (0.0)	0 (0.0)	-	0 (0.0)	0 (0.0)	-	0 (0.0)	0 (0.0)	-	**0 (0.0)**
**Last anal sex was with a __ during last sexual act:**													
Male	2 (7.7)	0 (0.0)	0.21	1 (1.4)	2 (3.8)	0.57	4 (28.6)	4 (25.0)	1.00	1 (1.4)	6 (7.6)	0.12	**20 (5.6)**
Female	0 (0.0)	1 (3.3)	1.00	0 (0.0)	0 (0.0)	N/A	1 (7.1)	1 (6.3)	1.00	8 (11.6)	1 (1.3)	0.01	**12 (3.4)**
**Last oral sex was with a __ during last sexual act:**													
Male	3 (11.5)	1 (3.3)	0.33	9 (12.7)	11 (21.2)	0.21	7 (50.0)	7 (43.3)	0.73	8 (11.6)	8 (10.1)	0.77	**54 (15.1)**
Female	1 (3.8)	8 (26.7)	0.03	16 (22.5)	19 (36.5)	0.09	5 (35.7)	7 (43.3)	0.65	19 (27.5)	9 (11.4)	0.01	**84 (23.5)**
^**+**^ **Number of sexual routes for sexual intercourse**													
One	16 (61.5)	24 (80.0)		58 (81.7)	32 (61.5)		11 (78.6)	10 (62.5)		40 (58.0)	56 (70.9)		**247 (69.2)**
Two	8 (30.8)	4 (13.3)		12 (16.9)	17 (32.7)		2 (14.3)	3 (18.8)		23 (33.3)	18 (22.8)		**87 (24.4)**
Three	2 (7.7)	2 (6.7)	0.28	1 (1.4)	3 (5.8)	0.03	1 (7.1)	3 (18.8)	0.73	6 (8.7)	5 (6.3)	0.26	**23 (6.4)**
**Number of current sexual partners**													
0	1 (3.8)	1 (3.3)		3 (4.2)	0 (0.0)		0 (0.0)	0 (0.0)		1 (1.4)	3 (3.8)		**9 (2.5)**
1	7 (26.9)	13 (43.3)		27 (38.0)	26 (50.0)		7 (50.0)	9 (56.3)		3 (4.3)	29 (36.7)		**121 (33.9)**
>1	4 (15.5)	5 (16.7)	0.85	22 (31.0)	26 (50.0)	0.25	7 (50.0)	7 (43.3)	1.00	11 (15.9)	12 (15.2)	0.004	**94 (26.4)**
No response	14 (53.8)	11 (36.7)		19 (26.8)	0 (0.0)		0 (0.0)	0 (0.0)		54 (78.3)	35 (49.4)		**133 (37.2)**
**Awareness about condom**													
Yes	14 (53.8)	23 (76.7)		63 (88.7)	49 (94.2)		8 (57.1)	9 (56.3)		57 (82.6)	46 (58.2)		**269 (75.3)**
No	12 (46.2)	7 (23.3)	0.07	8 (11.3)	3 (5.8)	0.35	6 (42.9)	7 (43.3)	0.96	12 (17.4)	33 (41.8)	0.001	**88 (24.6)**
**Knowledge of where to obtain condom**													
Yes	2 (7.7)	2 (6.7)		4 (5.6)	4 (7.7)		5 (35.7)	5 (31.3)		6 (8.7)	7(8.9)		**35 (9.8)**
No	24 (92.3)	28 (93.3)	1.00	67 (94.4)	48 (92.3)	0.65	9 (64.3)	11 (68.8)	0.80	63 (91.3)	72 (91.1)	0.97	**322 (90.2)**
^**+**^ **Use of condom at last sexual act**													
Anal	0 (0.0)	1 (3.3)	1.00	5 (7.0)	5 (9.6)	0.61	2 (14.3)	0 (0.0)	0.21	5 (7.2)	6 (7.6)	0.94	**24 (6.7)**
Oral	1 (3.8)	1 (3.3)	1.00	0 (0.0)	1 (1.9)	0.42	2 (14.3)	0 (0.0)	0.21	1 (1.4)	0 (0.0)	0.47	**6 (1.7)**
Vagina	16 (61.5)	14 (46.7)	0.27	41 (57.7)	14 (26.9)	0.001	7 (50.0)	4 (25.0)	0.16	35 (50.7)	34 (43.0)	0.35	**165 (46.2)**
**Reason for use of male condom**													
Prevent HIV/STI	2 (7.7)	1 (3.3)	0.59	9 (12.7)	0 (0.0)	*0.01	0 (0.0)	0 (0.0)	-	2 (2.9)	6 (7.6)	0.29	**20 (5.6)**
Prevent pregnancy	2 (7.7)	5 (16.7)	0.43	4 (5.6)	6 (11.5)	0.32	1 (3.3)	1 (6.3)	1.00	6 (8.7)	10 (12.7)	0.44	**35 (9.8)**
Prevent both	11 (42.3)	8 (26.7)	0.27	28 (39.4)	10 (19.2)	0.02	3 (10.0)	2 (12.5)	0.64	26 (37.7)	27 (34.2)	0.66	**115 (32.2**)
**Currently using female condom**													
Yes	0 (0.0)	3 (5.4)		4 (5.6)	0 (0.0)		1 (7.1)	0 (0.0)		3 (4.3)	1 (1.3)		**12 (3.4)**
No	26 (100.0)	27 (48.2)	0.24	67 (94.4)	52 (100.0)	0.14	13 (92.9)	16 (100.0)	0.47	66 (95.7)	78 (98.7)	0.34	**345 (96.6)**
^**+**^ **History of STI in last 12 months**													
Discharge	5 (19.2)	7 (12.4)	0.71	33 (46.5)	24 (46.2)	1.00	10 (71.4)	9 (56.3)	0.47	18 (20.1)	11 (13.9)	0.06	**117 (32.7)**
Itching	6 (23.1)	6 (10.7)	0.78	34 (47.9)	26 (50.0)	0.82	8 (57.1)	12 (75.0)	0.44	19 (27.5)	21 (26.6)	0.90	**132 (37.0)**
Sore	2 (7.7)	1 (1.8)	0.59	9 (12.7)	8 (15.4)	0.67	7 (50.0)	1 (6.3)	*0.02	8 (11.6)	3 (3.8)	0.11	**39 (10.9)**
**Knowledge of HIV transmission**													
Good	11 (42.3)	20 (35.7)		24 (33.8)	24 (46.2)		6 (42.9)	5 (31.3)		30 (43.5)	29 (36.7)		**149 (41.7)**
Poor	15 (57.7)	10 (17.9)	0.07	47 (66.2)	28 (53.8)	0.47	8 (57.1)	11 (68.7)	0.51	39 (56.5)	50 (63.3)	0.40	**208 (58.3)**
**Knowledge of HIV prevention**													
Good	14 (53.8)	21 (37.5)		35 (49.3)	35 (67.3)		5 (35.7)	8 (50.0)		31 (44.9)	34 (43.0)		**269 (73.4)**
Poor	12 (46.2)	9 (16.1)	0.21	36 (50.7)	17 (32.7)	0.05	9 (64.3)	8 (50.0)	0.43	38 (55.1)	45 (57.0)	0.82	**88 (24.6)**

^+^ Multiple responses possible.

#### Forms of sexual practice in the last 12 months of the survey

The majority of sexually active adolescents (95.2%) practiced vagina sex in the last 12 months of the survey. Also, 110 (30.8%) respondents practiced sexual intercourse through two or more routes. No respondent had practiced anal, oral and vagina sex in combination.Anal sex was practiced by 25 (7.0%) adolescents in the last 12 months (Table 3). There was no significant difference in the percentage of male adolescents who self-reported as HIV positive or HIV negative who practiced anal sex (13.0% vs 7.7%; p = 0.72). In addition, there was no significant difference in the percentage of female adolescents who self-reported as HIV positive or HIV negative who practiced anal sex (7.6% vs 6.7%; p = 1.00).

#### Forms of sexual practice at the last sexual act

Anal sex was practiced by 20 (5.6%) males and 12 (3.4%) females during the last sexual act preceding the survey. Male to male anal sex practice was reported by eight (2.2%) adolescents; male to female anal sex was reported by 21 (5.9%) adolescents, and female to female anal sex was reported by three (0.8%) adolescents. No heterosexual anal sex was reported among HIV negative adolescents. More than one in ten (11.6%) HIV positive males engaged in heterosexual anal sex. There was no significant difference in the percentage of HIV positive and HIV negative males who engaged in anal sex with females (11.6% vs 0.0%; p = 0.10).

Oral sex was practiced by 54 (15.1%) male and 84 (23.5%) female adolescents during their last sexual act. Significantly more female adolescents reported oral sex (23.5% vs 15.1%; p = 0.001). Male to male oral sex was reported by 27 (7.6%) adolescents while 43 (12.0%) adolescents reported female to female oral sex.

#### Multiple concurrent sexual partnerships

At the time of the survey, 348 (97.5%) sexually active adolescents had a sexual partner. Of these, 121 (34.8%) adolescents had only one sexual partner while 94 (27.0%) had more than one concurrent sexual partner. Significantly more female than male adolescents reported having a single sex partner (43.5% vs 24.4%; p <0.001). There was no significant difference in the percentage of adolescents who self-reported as HIV positive or HIV negative who had no sex partners at the time of this study (2.7% vs 3.6%; p = 0.67).

#### Condom awareness and condom use

The majority (75.3%) of currently sexually active adolescents were aware of condoms. There were however, significantly more females than male adolescents who self-reported as HIV positive who were not aware of condoms (41.8% vs 17.4%; p < 0.001). There was no difference in the percentage of female and male adolescents who self-reported as HIV negative who were not aware about condom (23.3% vs 46.2%; p = 0.07).

Few sexually active adolescents (9.8%) knew where to obtain condoms. Also, few adolescents used condom during their last anal (6.7%), oral (1.7%) or vaginal (46.2%) sex. More male than female adolescents who self-reported as untested used condom during their last vaginal sex (57.7% vs 26.9%; p = 0.001). The main reason for using condoms was to prevent pregnancy and HIV infection (32.2%). Significantly more male than female adolescents who self-reported as untested used condom during their last sexual act did so because of wanting to prevent HIV infection and pregnancy (39.4% vs 19.2%; p = 0.02).

#### Sexually transmitted infection symptoms in last 12 months of the survey

Also, 37% of respondents reported a STI symptom in the last 12 months of the survey. Ulcers were the least reported symptoms. More male than female adolescents who were known to be HIV positive but who self-reported as untested had genital ulcer (50.0% vs 6.3%; p = 0.02).

#### Sexually active adolescents and knowledge of HIV transmission and HIV prevention

Also, 58.3% of sexually active adolescents had poor knowledge of HIV transmission while 73.4% had good knowledge of HIV prevention. There were no significant sex differences in the percentage of sexually active adolescents who had good knowledge on HIV transmission and HIV prevention irrespective of the HIV status (Table 3).

### Predictors of use of condom during the last sexual act

Predictors of condom use during anal and oral sex could not be determined due to the low number of adolescents who practice anal and oral sexual intercourse during the last sexual act. The following variables increased the odds of using condom during the last vaginal intercourse by more than one fold: Being an adolescent 17 – 19 years old, being HIV negative, being HIV positive, engaging in oral sex in the last 12 months, engaging in sex with someone 10 years or more older, having genital discharge in the last 12 months, having genital ulcers in the last 12 months, having good knowledge about HIV transmission, and having good knowledge about HIV prevention. These increased odds were however, not statistically significant (Table 4).

**Table 4:** **Predictors of condom use by sexually active adolescents at last vagina intercourse using binomial logistic regression**

Predictors of condom use during last vagina sex act				
Variables	Unadjusted Odds ratio	Standard error	P value	Confidence interval
Age: 17 – 19 years	1.59	0.44	0.08	0.93 – 2.73
Female	0.43	0.11	0.001	0.26 – 0.70
Self-reported HIV negative status	1.31	0.48	0.46	0.64 – 2.70
Self-reported HIV positive status	1.15	0.32	0.61	0.67 – 2.00
Engaged in anal sex in last 12 months	0.18	0.12	0.009	0.05 – 0.65
Engaged in oral sex in last 12 months	1.42	0.44	0.26	0.77 – 2.62
Have sex with someone 10 years or more older	1.35	0.66	0.54	0.51 – 3.52
Know where to obtain a condom	0.73	0.19	0.24	0.44 – 1.23
Had genital discharge in last 12 months	1.47	0.45	0.20	0.82 – 2.67
Had genital itching in last 12 months	0.46	0.17	0.04	0.22 – 0.96
Had genital ulcers in last 12 months	1.56	0.47	0.14	0.87 – 2.83
Have good knowledge of HIV transmission	1.12	0.34	0.69	0.63 – 2.02
Have good knowledge of HIV prevention	1.80	0.63	0.09	0.91 – 3.57
Have concurrent sexual partnerships	0.39	0.20	0.07	0.14 – 1.08
_Cons	0.69	0.26	0.32	0.33 – 1.45

Being a female (p = 0.001), having engaged in anal sex in the last 12 months (p = 0.009), and having genital itch in the last 12 months (p = 0.04) significantly reduced the odds of using condom during the last vaginal sex.

## Discussion

We observed differences in sexual behaviour of adolescents based on sex and HIV status. Significantly more female adolescents who self-reported as HIV positive had experienced forced sexual initiation when compared to female adolescents who self-reported as HIV negative; more female than male adolescents had engaged in transactional sex; and more females than males had sex with partners who were 10 years older or more. The use of condoms during anal, oral and vagina sex was also very low. The odds of using a condom during the last vaginal sex act was reduced for females. Having a HIV positive or negative status did not significantly increase the odds of using a condom at last vaginal intercourse. More male than female adolescents who were known to be HIV positive but who self-reported as untested had genital ulcers. Having a history of genital itching during the last 12 months reduced the odds of using a condom during the last vagina sexual act. There were no differences in anal and vagina sexual practices based on sex and HIV status. However, significantly more females than males practice oral sex. Also, having a history of anal sex in the last 12 months reduced the odds of using a condom during the last vagina sexual act.

One of the strengths of the paper is that a national sample of adolescents was recruited for this study. The recruitment strategy increased the probability of including both in and out of school adolescents in the study sample. This is important as large numbers of adolescents in Nigeria are out-of-school[18]. Also, this is the first study to examine the sexual behaviour and sexual practices of a national sample of ALHIV in Nigeria. Nigeria has the world’s largest burden of mother to child transmission of HIV with only 30.1% coverage for its prevention of mother to children transmission of HIV programme[19]. The high perinatal transmission rate of HIV – put at 27.3% at the end of 2013[19] - and reduced rate of infant mortality from HIV due to successful antiretroviral programmes increase the likelihood of the country to have a teeming population of adolescents living with HIV. The study was also able to recruit adolescents younger than 15 years. This is also the first national adolescent survey that would conduct studies on the sexual practices and sexual behaviour of adolescents 10–14 years old in Nigeria. These make the findings of this study important for policy and programming for adolescents in Nigeria in general, and for ALHIV specifically.

Our study has its limitations. First, the study did not distinguish between adolescents who acquired HIV infection perinatally and those who acquire HIV behaviourally. Adolescents with sexually acquired HIV may differ substantially from adolescents with perinatal HIV exposure in that they are considerably older when first infected and have already engaged in sexual and/or drug risk behaviour leading to HIV infection[20, 21]. Lumping both groups of ALHIV without due consideration given to the behavioural differences during data analysis limits the generalizability of this finding to all ALHIV. However, the data collection process was limited in its ability to identify source of HIV infection from respondents since respondents needed to self-identify as HIV positive, negative or untested. As would have been observed in the data analysis, many of the adolescents whom we recruited as HIV positive either did not respond to the question or were not aware of their HIV status. Second, the study depended on self-reporting of HIV status introducing bias into the reporting of the HIV status of respondents. Third, a further bias was introduced into the study through the non-randomisation of the recruitment of study participants. This furthers limits the generalizability of the study findings. Fourth, the use of interviewer administered questionnaire may result in social desirability responses with further introduce bias. Despite these limitations, the study results provide insight into the sexual and reproductive health needs of adolescents 10 – 19 years old in Nigeria, especially the sexual and reproductive health needs of ALHIV.

One important finding was that more sexually active females who self-reported as HIV positive had experienced rape. This study can however, not show if HIV infection increases the risk for rape or rape increased the risk for HIV infection. This finding highlights a need to address rape among adolescents in Nigeria – its prevention and how to mitigate the risk of HIV infection following rape reports in view of the possibility that it may play an important role in increasing the risk for HIV infection in Nigeria. The high rate of forced sex reported among ALHIV is worth further investigation. It may be important to conduct this same study in perinatally infected children to be able to establish the relationship between rape, HIV infection and adolescence.

Also, the study reported that females, those who engaged in anal sex in the last 12 months, and those with a history of genital itching have significantly reduced odds of using a condom during sexual intercourse. The importance of these findings with respect to HIV infectivity cannot be underestimated. Females are worse affected by the HIV epidemic in Nigeria; the risk of HIV transmission through unprotected anal sex is very high – 18 times higher when compared with unprotected vagina intercourse[22]; and so also the risk of acquisition of HIV infection when there is an STI. The import of these findings for HIV infection in adolescents further becomes heightened due to evidence that shows that adolescents who engage in anal and oral sex also engage in more sexual risk behaviours such as having multiple sex partners and having sex without protection due to the erroneous belief of the safety of these practices[23]. It is therefore important to identify effective mechanisms to mitigate the risk for HIV infection and other STIs among adolescents in Nigeria.

One of such efforts would include increasing awareness about condom, its use, its benefits and where to access condoms for adolescents. The low level of awareness about condom and the low knowledge of where to buy a condom could have contributed to low use of condom during sex by study participants. However, little is known about how genital itching could have played a role in low use of condom during vaginal sex. It is also important to understand the association between genital itching and reduced odds of condom use especially when those with genital discharge and genital ulcers have increased odds of using condoms during vaginal sex. It is possible that there is the erroneous assumption that the genital itching may have resulted from the use of condom.

This study was not able to establish any difference in the practice of anal and vagina sex based on HIV status. Also, there was no difference in the percentage of adolescents who engage in sex with those 10 years older than them, and the percentage of adolescents who engaged in transactional sex based on HIV status. This study finding does not however, preclude that adolescent in Nigeria engage in risky sexual practices. Prior national studies have shown that adolescents in Nigeria do have a higher tendency to engage in high sexual risk behaviour – including unprotected sex during transactional sex – when compared to other age groups[3, 24].

Also important is the finding that more than a third of adolescents reported symptoms of STIs. Reports of STI symptoms were significantly higher among HIV positive males. Prior studies had established the relationship between STI and HIV infection with STIs being a recognized risk factor for HIV infection[25]. The finding that more male ALHIV gave a history of HIV symptoms further heightens the need for public education about diagnosis and appropriate management of STI infections especially for male adolescents.

The findings from this study have implication for policy formulation and programming for adolescents and ALHIV in Nigeria. First, the high prevalence of forced sexual initiation among adolescents needs to be addressed. The association found between HIV infection and a history of forced sexual initiation makes it important for the country to institute measures to address what can otherwise be called an epidemic. Measures also need to be instituted to enable adolescents learn about HIV prevention, transmission, condom use, where to access condom, and STI management. These measures are essential for a country like Nigeria with a huge HIV epidemic.

## Conclusion

There were differences observed in sexual practices and sexual behaviours of adolescents in Nigeria based on their sex. There is the need to better understand the drivers for non-use of condom among adolescents in Nigeria in a bid to increase its use by the population. It is also important to further explore the observed association between forced sexual initiation and HIV status in this study population to determine the direction of this association. This would help in planning rape prevention programmes for adolescents in Nigeria.

## References

[1] Patton G, Viner RM, Linh LC, Ameratunga S, Fatusi AO, Ferguson J, Patel V (2010). Mapping a global agenda for adolescent health. J Adolesc Health.

[2] Fatusi AO, Blum RB (2009). Adolescent health in an international context: the challenge of sexual & reproductive health in Sub-Saharan Africa. Adolesc Med State Art Rev.

[3] Federal Ministry of Health (2013). National HIV & AIDS and Reproductive Health Survey, 2012 (NARHS Plus).

[4] Birungi H, Mugisha JF, Nyombi J, Obare F, Evelia H, Nyinkavu H (2008). Sexual and reproductive health needs of adolescents perinatally infected with HIV in Uganda.

[5] Aboki H, Folayan MO, Daniel U, Ogunlayi M (2014). Changes in HIV sexual risk behaviour among adolescents aged 15–19 years over a five-year period - Is the HIV prevention programme in Nigeria yielding results?. Afr J Reprod Health.

[6] Van der Staten A, King R, Grinstead O, Vittinghoff E, Serufillira A, Allen S (1998). Sexual coercion, physical violence and HIV infection among women in steady relationships in Kigali, Rwanda. AIDS Behav.

[7] Jewkes RK, Dunkle K, Nduna M, Shai N (2010). Intimate partner violence, relationship power inequity, and incidence of HIV infection in young women in South Africa: a cohort study. Lancet.

[8] Ajuwon A, Adegbite O (2008). Ethical and methodological challenges involved in research on sexual violence in Nigeria. Res Ethics Rev.

[9] Akinyemi AO (2007). The analysis of records of sexual-related offences in Lagos state, Nigeria.

[10] Folayan MO, Brown B, Odetoyingbo M, Harrison A (2014). Rape in Nigeria: a silent epidemic among adolescents with implications for HIV infection. Glob Health Action.

[11] Federal Ministry of Health (2010). Technical report: 2010 national HIV sero-prevalence sentinel survey.

[12] Federal Ministry of Health (2008). National HIV/AIDS and Reproductive Health Survey, 2007 (NARHS Plus).

[13] National Agency for the Control of AIDS, Nigeria (2011). Joint Annual Review of the national response to HIV/AIDS.

[14] Yahaya LA, Jimoh AA, Balogun OR (2010). Factors hindering acceptance of HIV/AIDS voluntary counseling and testing (VCT) among youth in Kwara State, Nigeria. Afr J Reprod Health.

[15] Federal Ministry of Health (2005). National HIV/AIDS and Reproductive Health Survey, 2012 (NARHS Plus).

[16] Federal Ministry of Health (2008). HIV/STI integrated biological and behavioural surveillance survey (IBBSS).

[17] Federal Ministry of Health (2010). HIV/STI integrated biological and behavioural surveillance survey (IBBSS).

[18] UNICEF, Nigeria (2005). The children education report.

[19] National Agency for the Control of AIDS (2014). Global AIDS response country progress report.

[20] Koenig LJ, Pals SL, Chandwani S, Hodge K, Abramowitz S, Barnes W, D'Angelo L (2010). Sexual transmission risk behavior of adolescents with HIV acquired perinatally or through risky behaviors. J Acquir Immune Defic Syndr.

[21] Bauermeister JA, Elkington K, Brackis-Cott E, Dolezal C, Mellins CA (2009). Sexual behavior and perceived peer norms: comparing perinatally infected and affected youth. J Youth Adolesc.

[22] Baggaley RF, White RG, Boily MC (2010). HIV transmission risk through anal intercourse: systematic review, meta-analysis and implications for HIV prevention. Int J Epidemiol.

[23] Cherie A, Berhane Y (2012). Oral and anal sex practices among high school youth in Addis Ababa, Ethiopia. BMC Public Health.

[24] Federal Office of Statistics (2008). Nigeria Demographic and Health Survey (DHS).

[25] Piot P, Laga M (1989). Genital ulcers, other sexually transmitted diseases, and the sexual transmission of HIV. BMJ.

